# One‐Step Nucleic Acid Amplification Analysis of Sentinel Lymphatic Nodes in Endometrial Cancer Patients (EU‐OSNA): A European Multicenter Diagnostic Accuracy Study

**DOI:** 10.1002/cam4.71268

**Published:** 2025-09-22

**Authors:** Jan Kostun, Krzysztof Nowosielski, Marcin A. Jedryka, David Hardisson, Stefano Restaino, Sonia Gatius, Zoltan Novak, Amaia Sagasta Lacalle, Susana Lopez, Martin Pešta, Marcin Zebalski, Piotr Lepka, María Dolores Diestro, Giuseppe Vizzielli, Xavier Matias‐Guiu, Tímea Echim, Emma Natalia Camacho Urkaray, Iván Rienda, Robert Slunečko, Andrzej Czekanski, Alberto Berjón, Laura Mariuzzi, Ana Velasco, Judit Betenbuk, Isabel Guerra Merino, Pablo Padilla‐Iserte, Petr Stráník, Vendula Smoligová, Jiří Presl

**Affiliations:** ^1^ Department of Gynecology and Obstetrics University Hospital in Pilsen, Charles University Pilsen Czech Republic; ^2^ Department of Gynecology and Gynecological Oncology University Clinical Center of Medical University of Silesia Katowice Poland; ^3^ Department of Gynecology and Gynecological Oncology District Teaching Hospital Czeladz Poland; ^4^ Department of Oncological Gynecology Lower Silesian Oncology, Pulmonology and Hematology Center Wroclaw Poland; ^5^ Department of Gynecological Oncology Wroclaw Medical University Wroclaw Poland; ^6^ Department of Pathology Hospital Universitario La Paz Madrid Spain; ^7^ Molecular Pathology and Therapeutic Targets Group Hospital La Paz Institute for Health Research (IdiPAZ) Madrid Spain; ^8^ Center for Biomedical Research in the Cancer Network (Centro de Investigación Biomédica en Red de Cáncer, CIBERONC), Instituto de Salud Carlos III, Faculty of Medicine Universidad Autónoma de Madrid Madrid Spain; ^9^ Clinic of Obstetrics and Gynecology ‘Santa Maria della Misericordia’ University Hospital, Azienda Sanitaria Universitaria Friuli Centrale Udine Italy; ^10^ Department of Pathology Hospital Universitari Arnau de Vilanova de Lleida, Oncological Pathology Group, IRBLleida, Universitat de Lleida, CIBERONC Lleida Spain; ^11^ Department of Gynecology National Institute of Oncology Budapest Hungary; ^12^ Department of Pathology Hospital Universitario Araba, Osakidetza, Instituto de Investigación Sanitaria Bioaraba, Universidad del País Vasco UPV/EHU Vitoria‐Gasteiz Spain; ^13^ Department of Pathology Hospital Universitario y Politecnico La Fe Valencia Spain; ^14^ Faculty of Medicine in Pilsen, Institute of Biology Charles University Pilsen Czech Republic; ^15^ Gynecologic Oncology Unit, Department of Gynecology and Obstetrics Hospital Universitario La Paz Madrid Spain; ^16^ Hospital La Paz Institute for Health Research (IdiPAZ), Faculty of Medicine Universidad Autónoma de Madrid Madrid Spain; ^17^ Department of Medicine University of Udine Udine Italy; ^18^ Sikl's Department of Pathology University Hospital in Pilsen, Charles University Pilsen Czech Republic; ^19^ Institute of Pathological Anatomy ‘Santa Maria della Misericordia’ University Hospital, Azienda Sanitaria Universitaria Friuli Centrale Udine Italy; ^20^ Department of Pathology National Institute of Oncology Budapest Hungary; ^21^ Department of Gynecologic Oncology Hospital Universitario y Politecnico La Fe Valencia Spain

**Keywords:** benign epithelial inclusions, endometrial cancer, OSNA, sentinel node, ultrastaging

## Abstract

**Objective:**

This European multicenter study aimed to assess the diagnostic accuracy of one‐step nucleic acid amplification (OSNA) as the primary endpoint by comparing this method with ultrastaging for the detection of sentinel node metastases in endometrial cancer patients.

**Methods:**

European multicenter prospective performance study including data from 10 centers across 5 European countries. Each node, upon removal of surrounding adipose tissue, was sliced in 2 mm thick sections and equally distributed between ultrastaging and OSNA. OSNA is based on cytokeratin‐19 detection, serving as a metastatic marker. Sensitivity, specificity, and concordance of OSNA versus ultrastaging were calculated at nodal and patient levels.

**Results:**

Seven hundred forty‐three sentinel nodes from 366 patients were evaluated. Compared to ultrastaging, OSNA showed concordance, specificity, and sensitivity of 95%, 97.6%, and 41.2% at the nodal level and 93.2%, 96.2%, and 47.8% at the patient level, respectively. In reverse analysis, when compared to OSNA, the ultrastaging showed a sensitivity of 45.2% and 45.8% at the nodal and patient levels, respectively. Irrespective of the size of metastasis, both methods agreed in 14 positive and 692 negative nodes (95%). This resulted in 24 (6.56%) patients with a positive OSNA and 23 (6.28%) patients with a positive ultrastaging finding. The number of discordant nodes was 47 (6.33%), 40 (85.1%) of them were micrometastases. Benign epithelial inclusions occurred in 4 nodes (0.54%) and 4 patients (1.09%).

**Conclusion:**

Compared with ultrastaging, OSNA showed high concordance and specificity, but sensitivity was low—similar to ultrastaging compared with OSNA as an index test in reverse analysis. The main limitation in comparing the two approaches by splitting the sentinel nodes was the tissue allocation bias. As reflected in the number of discordant cases, especially at the micrometastases level. The distribution of patients with node metastases was comparable between the two methods at both the nodal and patient levels.

**Trial Registration:**

German Clinical Trial Register: Nr. DRKS00021520

## 1 Introduction

Endometrial cancer (EC) contributes to more than 124,000 new cases and about 30,000 deaths per year in Europe alone, and its incidence continues to rise [1, 2].

In recent years, the sentinel lymph node (SLN) concept has become an integral part of the management in patients with incipient EC. Its detection and detailed histopathological examination by ultrastaging has become a key component of surgical staging [3]. However, an expert pathologist is required as the differentiation of individual histological tumor subtypes and the ultrastaging examination of the SLN are challenging [4, 5]. Additionally, the SLN ultrastaging protocol is not standardized and varies between centers [6].

Hence, there is a need for a widely applicable, accurate, reproducible, and standardizable method for EC surgical staging. The molecular method utilizing one‐step nucleic acid amplification (OSNA) has been proposed as a suitable alternative. A major advantage of OSNA over ultrastaging lies in its high degree of automation and standardization, thus minimizing observer variability and reducing reliance on interpretive histopathological skills. Another reason for adopting OSNA is also its capacity for real‐time, intraoperative evaluation of SLNs. Unlike ultrastaging, which is labor‐intensive and requires multiple serial sectioning and immunohistochemical (IHC) staining steps that may take days.

OSNA is based on the detection of cytokeratin 19 mRNA (CK 19 mRNA) in lymphatic tissue upon its homogenization (whole node or its parts where applicable). CK 19 is not expressed within a healthy lymphatic tissue, and its presence in the lymph node indicates metastatic involvement. The reverse transcription loop‐mediated isothermal amplification (RT‐LAMP) is used to detect the exact number of copies of CK 19 mRNA in the specimen. The availability of results within approximately 30 min enables the intra‐operative use of the method.

The OSNA assay has been extensively validated for intraoperative detection of SLN metastases in breast cancer and has been integrated into clinical practice in several countries, including Japan, Germany, and the United Kingdom. Pivotal studies employing similar methodology to that used in our investigation have demonstrated the diagnostic accuracy of OSNA [7, 8]. Subsequent large‐scale studies have confirmed these findings and further demonstrated the clinical utility of OSNA in the management of breast cancer patients [9, 10, 11].

Recently, different studies have examined the utilization of OSNA for the detection of lymph node macro‐ and micro‐metastases in EC patients and compared this method to standard histopathology and ultrastaging [12, 13, 14, 15]. However, these studies were either conducted on a previous generation OSNA analyzer (RD 100i) or on a limited cohort size. Thus, more robust data were needed to evaluate the use of OSNA in clinical practice in patients with EC.

The aim of this prospective European multicenter study (EU—OSNA) was to assess the diagnostic accuracy of OSNA, as the primary endpoint, for the detection of metastases in SLNs in EC patients, using histopathological ultrastaging as the reference standard.

## 2 Methods

### 2.1 Study Protocol

This was a prospective multicenter diagnostic accuracy study involving 10 oncology centers in 5 European countries. The study was conducted in accordance with ethical principles of the Declaration of Helsinki, Good Clinical Practice, and any regional or national regulations of participating sites. Potentially eligible patients received information about the study and were only enrolled if they provided a valid written informed consent.

Inclusion criteria of the study comprised women aged ≥ 18 years with a diagnosis of EC, irrespective of histological subtype, who were scheduled for surgical treatment including sentinel lymph node mapping (SLNM) and had provided signed informed consent. Patients were not enrolled if informed consent was not obtained or if SLNM was unsuccessful. Preoperative imaging and the indication for SLNM were conducted according to routine clinical practice at each participating oncology center and were not restricted by the study design.

The recruitment target was a minimum of 300 patients. Patients meeting the study inclusion criteria underwent surgery between August 2020 and November 2022 at the following participating centers: University Hospital in Pilsen (Czech Republic; principal investigator site), University Hospital in Katowice (Poland), Hospital Czeladz (Poland), Lower Silesian Oncology, Pulmonology and Hematology Center in Wroclaw (Poland), University Hospital La Paz (Madrid, Spain), Azienda Sanitaria Universitaria Friuli Centrale (Udine, Italy), University Hospital Arnau de Vilanova (Lleida, Spain), National Institute of Oncology (Budapest, Hungary), Hospital Universitario Araba (Vitoria‐Gasteiz, Spain), and Hospital Universitario y Politecnico La Fe (Valencia, Spain). All the study data were collected in the central REDCap electronic database.

Patients underwent pre‐operative examination and subsequent surgical treatment in line with the participating center's local protocol. Any surgical approach was accepted, including laparoscopic, robot‐assisted, or open laparotomy.

All SLNs were examined simultaneously by OSNA (index test) and histopathological ultrastaging (gold standard test). Further clinical management was based on the results of ultrastaging in line with current international recommendations [16].

### 2.2 Sentinel Lymph Node Mapping and Processing

SLNM via superficial (1–3 mm) and deep (10–20 mm) intracervical injection of the tracer was performed according to the NCCN algorithm for tracer application [17, 18, 19]. Indocyanine green (ICG) was recommended as the SLNM method due to its demonstrated high reliability and identification rate of SLNs per hemipelvis in comparison to other techniques [17, 20, 21, 22]. Nonetheless, participating centers' dye of choice was also accepted.

**FIGURE 1:**
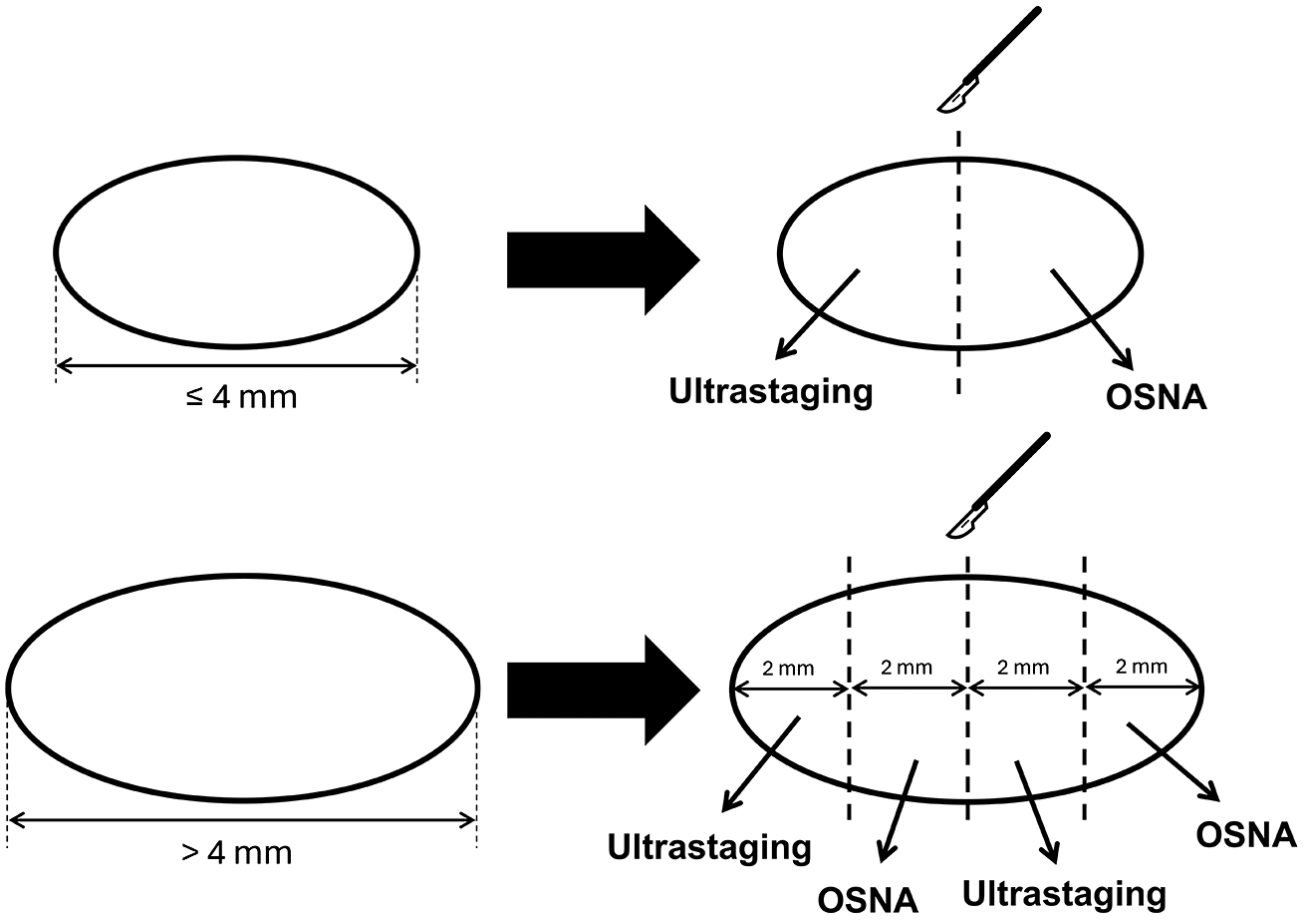
Sentinel lymph node—tissue allocation to OSNA and ultrastaging.

The number and anatomical location of harvested SLNs was recorded in the electronic REDCap database. Under sterile conditions, nodes were mechanically cleaned from fatty tissue to prevent contact with any epithelium and serially sectioned at 2 mm thickness slices perpendicular to the longest axis of the node. Odd and even sections were sent for OSNA analysis and histopathological ultrastaging, respectively. SLNs of 4 mm and less in their longest axis were cut in halves, one half attributed to each method (OSNA and ultrastaging). See Figure 1.

### 2.3 Ultrastaging

Slices for ultrastaging were fixed in 10% buffered formalin, embedded in a paraffin block, and submitted to the pathologist. From each paraffin block, levels at 150 μm intervals were sliced. At each level, three adjacent sections were cut, and the first section of each level was stained with hematoxylin and eosin (H&E). The second section was stained with immunohistochemistry (IHC) using the anti‐cytokeratin AE1:AE3 to detect low volume metastatic disease. The third was kept as a spare section to be used for repeat staining if required. If no micro‐ or macrometastasis was detected, the SLN was considered negative. If a metastasis was considered to be > 0.2 mm, the SLN was considered positive (> 2.0 mm = macrometastasis; > 0.2 mm but ≤ 2.0 mm = micrometastasis). The finding of isolated tumor cells (ITCs; clusters of tumor cells ≤ 0.2 mm) was documented, but the node was considered as negative [23].

### 2.4 OSNA Analysis

OSNA analysis was performed according to the manufacturer's instructions. SLN sections dedicated to OSNA analysis were placed in sterile biopsy sample containers, immediately refrigerated (2°C–8°C) or stored on ice (0°C–4°C) without drying to avoid RNA degradation and tightly fastened to avoid tissue dehydration. Recommended transport time between surgery and the laboratory was within 15 min in all study centers. If OSNA could not be performed within 8 h following node resection, samples were stored at −80°C. The weight of OSNA samples for homogenization was acceptable within a range of 25–600 mg. For samples exceeding 600 mg, a subdivision of the tissue into sub‐samples was mandatory. Since the OSNA method can process the whole lymph node, slices from the same node were pooled together into one vial for storage and further analysis purposes.

Frozen samples were homogenized in 4 mL of lysing buffer, as per the manufacturer's instructions (Sysmex, Kobe, Japan). LYNOPREP Blade Set was used for centrifugation. The lysate was then processed in the OSNA analyzer RD‐210, where the isothermal amplification of CK 19 mRNA by RT‐LAMP was performed. SLNs were defined as “negative” or “positive” according to established cut‐off values adopted from breast cancer metastasis description. SLNs were classified as negative for CK 19 mRNA with fewer than 250 copies/μL, classified as positive for micrometastases (+) when mRNA CK 19 levels were between 250 and 4999 copies/μL and with macrometastases (++) when the CK 19 mRNA level was 5000 mRNA copies/μL or more [7, 24].

### 2.5 Primary Tumor Examination

The immunohistochemical detection of CK19 was performed in every primary uterine tumor to confirm its expression (any positivity was taken into account).

### 2.6 Statistical Analysis

The description of the study population was done through quantitative summary using mean, median, absolute counts, and percentages.

The main goal of our analysis was to describe the sensitivity, specificity, positive and negative predictive values, and concordance of the OSNA method compared to ultrastaging. A reverse data analysis was also performed with OSNA as the standard and ultrastaging as the index test.

The primary endpoint was diagnostic accuracy at the nodal level. Patient‐level diagnostic performance was also evaluated by cluster analysis of SLNs data belonging to the same patient.

### 2.7 Quality Assurance

For quality assurance purposes, submission of all specimens and full pathology reports was required at least from two patients per center (randomly selected by the Principal Investigator) and reviewed centrally at Sikl's Department of Pathology, University Hospital Pilsen, Charles University, Czech Republic. Additional cases were reviewed if a major deviation from the study protocol was identified.

In accordance with the journal's guidelines, we will provide our data for independent analysis by a selected team by the Editorial Team for the purposes of additional data analysis or for the reproducibility of this study in other centers if such is requested.

## 3 Results

**FIGURE 2:**
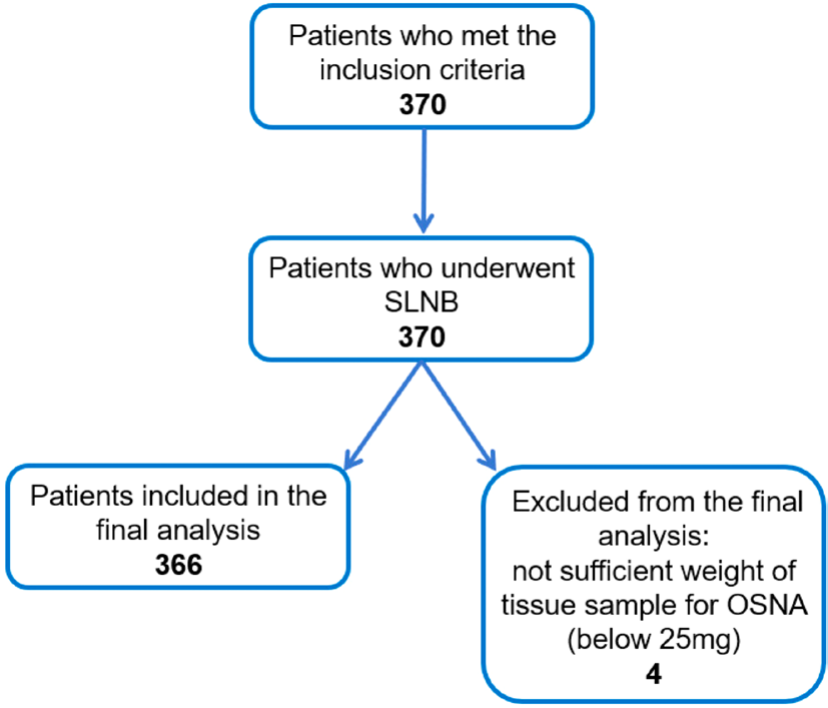
Patient accrual—flow diagram.

A total of 366 patients were recruited into the study between August 2020 and November 2022 (Figure 2), and a total of 743 SLNs were examined.

SLNM was successful bilaterally in 313 (85.52%) and unilaterally in 53 (14.48%) patients. ICG was applied as a tracer in 364 (99.45%) of them. Blue dye and magnetic tracer were used in one patient each. Systematic pelvic ± para‐aortic lymphadenectomy and SLN detection were performed in 32 (8.75%) patients. Patient characteristics are presented in Table 1.

**TABLE 1:** Patients characteristics.

Characteristics	Patients *n* = 366
Age (years)—median (min–max)	64 (29–87)
BMI (kg/m^2^)—median (min–max)	29.7 (18.6–58.2)
*Preoperative TNM staging*	
T—*n* (%)	
T0	3 (0.82)
T1a	240 (65.57)
T1b	105 (28.69)
T2	10 (2.73)
T3a	6 (1.64)
T3b	1 (0.27)
T4	1 (0.27)
N—*n* (%)	
NX	3 (0.82)
N0	359 (98.09)
N1	4 (1.09)
M—*n* (%)	
M0	363 (99.2)
MX	3 (0.8)
Pathological FIGO stage^a^—*n* (%)	
IA	174 (47.54)
IB	93 (25.41)
II	38 (10.38)
IIIA	12 (3.28)
IIIB	21 (5.74)
IIIC1	24 (6.56)
IIIC2	3 (0.82)
IVA	1 (0.27)
Tumor grade—*n* (%)	
Grading 1	151 (41.26)
Grading 2	164 (44.81)
Grading 3	51 (13.93)
Histological type—*n* (%)	
Endometrioid carcinoma	344 (93.99)
Serous carcinoma	10 (2.73)
Clear cell carcinoma	5 (1.37)
Dedifferentiated carcinoma	3 (0.82)
Carcinosarcoma	2 (0.55)
Mixed	2 (0.54)
LVSI—*n* (%)	
L0	268 (73.22)
L1	93 (25.41)
Unknown	5 (1.37)

Abbreviations: BMI, body mass index; LVSI, lymphovascular space invasion.

^a^ FIGO—International Federation of Gynecology and Obstetrics, version from 2009, stage based on postoperative histological results.

SLNs' longest mean axis length, mean weight, and thickness were 14.19 mm (7.19), 240.82 mg (281.26), and 2.15 mm (0.47), respectively. Further SLNs' characteristics as well as their histopathological and OSNA examination findings are presented in Table 2.

**TABLE 2:** Sentinel lymph node characteristics.

Histopathological and OSNA characteristics of the sentinel lymph nodes		
Characteristics	SLNs *n* = 743	Patients *n* = 366
*Histopathology*		
SLN tumor involvement—*n* (%)		
Tumor free	702 (94.48)	337 (92.08)
ITCs	7 (0.94)	6 (1.64)
Micrometastasis	22 (2.96)	13 (3.55)
Macrometastasis	12 (1.62)	10 (2.73)
SLN tumor involvement—*n* (%)		
Negative	709 (95.42)	343 (93.72)
Micrometastasis	22 (2.96)	13 (3.55)
Macrometastasis	12 (1.62)	10 (2.73)
SLN metastasis size (mm)		
*N*	34	23
Mean (SD)	3.45 (6.33)	4.35 (7.46)
Median (min‐max)	1.10 (0.20–35.00)	1.30 (0.20–35.00)
SLN benign epithelial inclusion—*n* (%)		
No	739 (99.46)	362 (98.91)
Yes	4 (0.54)	4 (1.09)
*OSNA results*		
OSNA result—*n* (%)		
Negative [< 160 copies/μL]	708 (95.29)	338 (92.35)
(−)L [160–249 copies/μL]	4 (0.54)	4 (1.09)
Positive—Micrometastasis [250–4999 copies/μL]	24 (3.23)	18 (4.92)
Positive—Macrometastasis [≥ 5000 copies/μL]	7 (0.94)	6 (1.64)
OSNA result—*n* (%)		
Negative [≤ 249 copies/μL]	712 (95.83)	342 (93.44)
Micrometastasis [250–4999 copies/μL]	24 (3.23)	18 (4.92)
Macrometastasis [≥ 5000 copies/μL]	7 (0.94)	6 (1.64)
OSNA (CK19 mRNA copies/μL)		
Mean (SD)	1108.76 (21,664.50)	2250.85 (30,891.44)
Median (min–max)	0.00 (0.00–580,000.00)	0.00 (0.00–580,000.00)

All 366 primary tumors were immunohistochemically tested for the expression of CK 19 protein. Its expression was confirmed in 100% of tested tumors regardless of their histology, with a mean CK 19 expression of 77.30% (SD 23.85%) and a median of 80% (interquartile range [IQR] 30). The mean tumor size was 29.7 mm (SD 1.74) with a median of 27.0 mm (1–100 mm).

We focused on comparing the results of both methods not only at the nodal level but also at the patient level. OSNA showed a concordance rate with histopathological examination of 95% and 93.2% at the nodal and patient levels, respectively (Table 3).

**TABLE 3:** Results consistency.

Consistency between OSNA and pathological ultrastaging—patient level				
At patient level		Ultrastaging		
		Positive	Negative	Total
OSNA	Positive	11	13	24
	Negative	12	330	342
	Total	23	343	366
Concordance		93.20% (90.11–95.33)		
Sensitivity		47.80% (29.24–67.04)		
Specificity		96.20% (93.62–97.77)		
False negatives		52.20% (47.08–57.32)		
False positives		3.80% (1.84–5.76)		
Reverse sensitivity		45.80% (27.89–64.93)		
Reverse specificity		96.50% (93.97–97.98)		
Kappa index		0.4342 (0.3834–0.485)		
*p*‐value (McNemar)		1		

Consistency between OSNA and pathological ultrastaging—nodal level				
At node level		Ultrastaging		
		Positive	Negative	Total
OSNA	Positive	14	17	31
	Negative	20	692	712
	Total	34	709	743
Concordance		95.00% (93.21–96.37)		
Sensitivity		41.20% (26.37–57.78)		
Specificity		97.60% (96.19–98.5)		
False negatives		58.80% (55.26–62.34)		
False positives		2.40% (1.3–3.5)		
Reverse sensitivity		45.20% (29.16–62.23)		
Reverse specificity		97.20% (95.7–98.17)		
Kappa index		0.4024 (0.3671–0.4377)		
*p*‐value (McNemar)		0.7428		

Ultrastaging detected a total of 22 (2.96%) micrometastases and 12 (1.62%) macrometastases. Among the negative results, there were 7 (0.94%) cases with isolated tumor cells (ITCs). OSNA detected a total of 24 (3.23%) micrometastases and 7 (0.94%) macrometastases. In 4 (0.56%) cases of negative results, the level of CK19 mRNA copies was just below the threshold of negativity, that is, between 160 and 249 copies/μL. However, both methods agreed only in one case of detected macrometastases and in 3 cases of micrometastases. Regardless of the size of the metastasis, agreement between the two methods occurred in 14 positive and 692 negative SLNs—95% (Table 4).

**TABLE 4:** Discrepant results chart—nodal level.

Results distribution between OSNA and pathological ultrastaging at the SLN level						
*n* = 743		Ultrastaging				
		Tumor free	ITCs	Micro‐metastasis	Macro‐metastasis	Total
OSNA	Negative [< 160 copies/μL]	685	5	15	3	708
	Negative (−)L [160–249 copies/μL]	2	0	0	2	4
	Micrometastasis [250–4999 copies/μL]	13	2	3	6	24
	Macrometastasis [≥ 5000 copies/μL]	2	0	4	1	7
	Total	702	7	22	12	

*Note:* OSNA results—Cytokeratin 19 mRNA copies/μL.

Abbreviations: ITCs, isolated tumor cells; SLN, sentinel lymph node.

## 4 Discussion

### 4.1 Summary of Main Results

In this largest European multicenter prospective study, we analyzed the diagnostic accuracy of OSNA in comparison to histopathological ultrastaging of SLN in patients with EC undergoing surgical treatment.

In our patient cohort, OSNA had 95% concordance, 97.6% specificity, and 41.2% sensitivity compared to histopathological ultrastaging at a nodal level and 93.2% concordance, 96.2% specificity, and 47.8% sensitivity compared to histopathological ultrastaging at the patient level. In contrast, when OSNA was set as the standard approach, ultrastaging had a reverse sensitivity of 45.2% and 45.8% at the nodal and patient levels, respectively. The total number of metastatic nodes detected by OSNA was 31 and by ultrastaging 34, while the difference at the patient level was 24 versus 23 nodal positive patients, respectively.

### 4.2 Results in the Context of Published Literature

In terms of concordance, specificity, and sensitivity, our results concur with La Fera et al. on a similarly sized nodal sample (668 SLNs) [13]. Their work described a high concordance (96.7%) and specificity rate (98.4%). Since they also analyzed different nodal portions using OSNA and ultrastaging, they observed a high number of discordant nodes (3.3%), which resulted in a sensitivity of 50%. In our case, the even lower sensitivity for both approaches was related to a greater portion of discrepant cases, 47 out of 743 (6.33%).

In a multicenter study published in 2021 by Diestro et al., 526 sentinel lymph nodes from 191 patients were analyzed using a distinct tissue allocation protocol. Specifically, a 1 mm central slice of each node was examined by ultrastaging, while the remaining tissue was subjected to OSNA. This approach led to a higher detection rate of metastases by OSNA, resulting in 13.6% discordant cases and the upstaging of 8.2% of patients. The increased detection by OSNA may be partly attributed to tissue allocation bias, as the majority of each lymph node was analyzed by OSNA rather than by ultrastaging [14].

In other previously published papers, the sensitivity of OSNA was reported to be significantly higher, up to 100%. High sensitivity values were achieved mainly in monocentric studies conducted on a significantly smaller population sample. In such cases, the issue of tissue allocation bias seems to be less pronounced, possibly also due to a reduced intra‐sample variation in the thickness of the SLN slices [12, 25, 26, 27].

**FIGURE 3:**
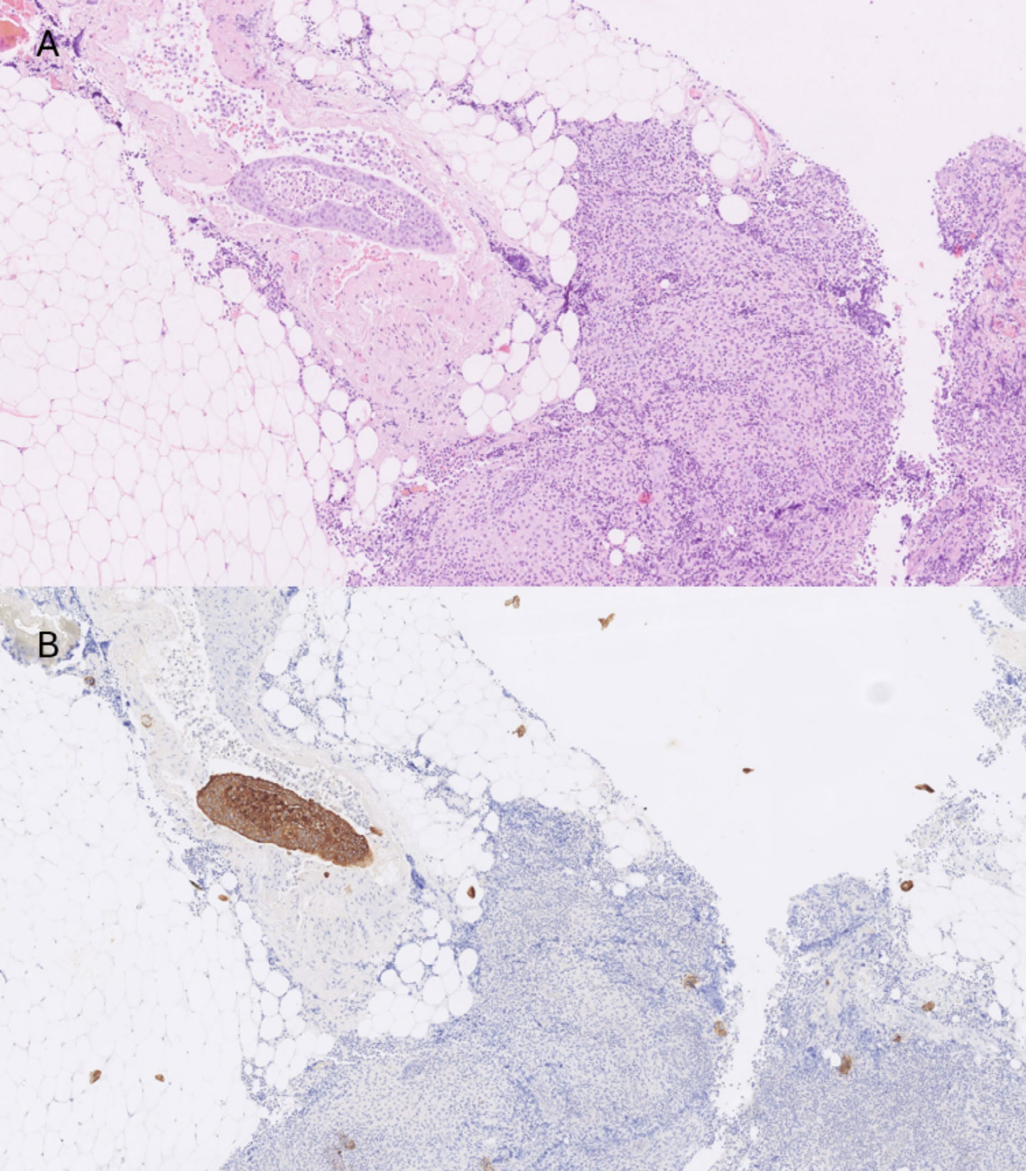
Micrometastasis at the sentinel lymph node margin in an OSNA‐negative, ultrastaging‐positive discrepant case. Representative histological images showing micrometastasis located within a vessel at the periphery of a lymph node. (A) Hematoxylin and eosin (H&E) staining. (B) Immunohistochemical staining using cytokeratin AE1/AE3.

In our OSNA results, there were 15 cases of micrometastases and 2 cases of macrometastasis with a negative ultrastaging result. While on the other hand, there were OSNA‐negative SLNs with positive ultrastaging results for micrometastases and macrometastasis in 15 and 5 cases, respectively. The median size of the metastasis found in the OSNA‐negative discordant SLNs was 0.8 mm (0.2–6), while in the ultrastaging‐negative SLNs, a median value of 810 CK‐19 mRNA copies/μL (300–28,000) with OSNA was detected, corresponding to micrometastases (Figure 3 representative histological images of a discordant case). This further confirms that in the case of small nodal tumor burden, analyzing different tissue slices from the same tissue with two different approaches may lead to an inequity in the sample's distribution and to an elevated discordancy rate.

This discrepancy was reported to result in changing patients' FIGO stage [28]. Indeed, it was reported that 11 (3%) patients with FIGO I and 2 (0.55%) patients with FIGO II would have been upstaged to FIGO III based on OSNA. In comparison, 12 (3.3%) patients from FIGO III according to ultrastaging would have been downstaged by OSNA to FIGO I (9 patients), FIGO II (2 patients), and 1 patient would have been classified as FIGO IIIB instead of FIGO IIIC1.

### 4.3 Strengths and Weaknesses

To our knowledge, this is the largest study analyzing the clinical performance of OSNA in EC patients to date. The study took place in 10 European centers over a period of 3 years and enrolled a total of 366 patients and examined 743 SLNs.

The biggest weaknesses of our study are the relatively low proportion of positive nodes and the risk of tissue allocation bias resulting from the inability to test the same sample of tissue using both methods. However, reverse analysis of the data with OSNA as the standard method showed a similar distribution of results with reverse sensitivity and reverse specificity of 45.2% and 97.2% at the nodal level and 45.8% and 96.5% at the patient level. According to our study, both methods, ultrastaging and OSNA, produced a comparable proportion of metastatic lymph node involvement in the study population.

The current generation OSNA analyzer (RD‐210) used in our study is CE‐certified and fully compliant with Directive 98/79/EC. It is validated for use in gynecological malignancies, expanding beyond the scope of the previous model (RD‐100), which was primarily applied in breast, colorectal, and gastric cancers. Compared to its predecessor, the RD‐210 offers several technical advancements: it can process up to 14 samples simultaneously, features a shorter amplification time, and includes automated functionalities—such as reagent and sample management and data tracking—that were previously performed manually (www.sysmex‐europe.com).

Since different parts of the lymph node are used for the index and reference tests, the performance indicators described in the studies published so far, this study included, cannot be considered entirely precise. The correct use of the terms “sensitivity” and “specificity” would require the analysis of identical pieces of the lymph node with both methods.

In 2007, Tsujimoto et al. published an original study that established CK19 as the optimal marker for the OSNA detection of lymph node metastases in breast cancer [7]. Among 45 candidate mRNA markers identified from the EST database [29], CK19 was selected due to its high expression in metastatic tissue and minimal or absent expression in normal lymphatic tissue. Similar results were published by Nagai et al. in 2015 for endometrial cancer, where 24 potential mRNA markers were evaluated [26]. Among these, CK19, CK7, and EpCAM demonstrated the greatest utility for detecting nodal metastases. Ultimately, CK19 was identified as the most suitable molecular target. Nevertheless, ongoing advances in molecular biology may reveal alternative or complementary biomarkers with improved sensitivity and specificity in the context of nodal metastasis detection in EC.

The 2 mm sectioning interval of SLNs employed in this study was adopted from a pilot study by Kostun et al., which compared OSNA assay to histopathological ultrastaging as the reference standard in EC [12]. This single‐center study demonstrated that 2 mm slicing enabled comparison between the two diagnostic modalities and showed OSNA was capable of detecting a clinically relevant number of micrometastases relative to ultrastaging. The findings of that pilot study supported the feasibility and diagnostic validity of the 2 mm sectioning protocol, which was therefore implemented in the present multicenter study. Despite its practical advantages, the 2 mm interval protocol may limit the detection of very small micrometastases and isolated tumor cells due to allocation bias. Failure to detect nodal metastatic involvement may adversely impact postoperative management and, consequently, clinical outcomes.

### 4.4 Implication for Practice and Future Research

Our findings indicate that the slice thickness of 2 mm used in our study protocol and the variance of its thickness measured during the study (mean 2.15 mm (SD 0.47)) may have resulted in allocation bias, particularly in cases of micrometastatic SLN involvement. This potential limitation highlights the need for further optimization of OSNA node processing study protocols in future research.

The overall incidence of benign epithelial inclusions in our study was low and does not appear to represent a substantial limitation to the clinical use of the OSNA method. Among the 743 SLNs analyzed, only 4 nodes (0.54%) with benign epithelial inclusions were identified by ultrastaging. In none of these cases did the presence of benign epithelial inclusions affect the overall nodal assessment or alter FIGO staging. In contrast, other studies in EC have reported a higher occurrence of benign epithelial inclusions in SLNs. For instance, La Fera et al. found benign epithelial inclusions in 17 of 668 SLNs (2.5%), which contributed to one OSNA false‐positive result. Similarly, in the study by López‐Ruiz et al., two out of 94 SLNs (2.1%), both from the same patient, were identified as OSNA false‐positives due to CK19‐positive benign epithelial inclusions. Although a rare phenomenon, the potential impact of benign epithelial inclusions on OSNA specificity justifies further investigation [13, 25].

The relatively low sensitivity of the OSNA assay observed in our study raises important clinical considerations, particularly regarding the risk of false‐negative results. Such outcomes may lead to the failure to detect micrometastatic or even macrometastatic disease. This concern is especially pertinent in patients with EC, where accurate assessment of lymph node involvement is critical for determining appropriate adjuvant therapy. In this context, the risk of disease understaging and consequent undertreatment may outweigh the advantages of a rapid and standardized diagnostic approach. Therefore, further methodological refinement and prospective validation of the OSNA technique are warranted to enhance its diagnostic accuracy.

Given that the same tissue sample cannot be analyzed simultaneously by both OSNA and ultrastaging, we believe that future studies should be designed to mitigate allocation bias through methodological strategies. One such approach could involve the complete allocation of entire SLNs to either OSNA or ultrastaging in a randomized manner within a large, prospective, multicenter trial. This design would allow for an unbiased comparison of diagnostic performance at the patient level while preserving tissue integrity for each method.

## 5 Conclusions

OSNA demonstrated high concordance and specificity compared to SLN histopathological ultrastaging in patients with endometrial cancer. The lower sensitivity observed in this study may be partially attributed to tissue allocation bias, as it is not feasible to analyze the exact same nodal tissue using both methods. Nevertheless, OSNA demonstrated a comparable distribution of positive and negative findings to that of ultrastaging, supporting its reliability as a diagnostic tool within the studied cohort.

Importantly, while OSNA is not without limitations, it offers a standardized, rapid, and automatable approach to lymph node assessment. This represents a significant advancement over conventional histopathology, which remains labor‐intensive and time‐consuming. To mitigate the impact of sampling bias and further validate OSNA's diagnostic performance, future investigations should employ randomized whole node allocation strategies and be conducted within large, multicenter cohorts. Such studies are essential to solidify the role of OSNA in routine clinical staging algorithms.

## Author Contributions

**Jan Kostun:** conceptualization, investigation, writing – original draft. **Krzysztof Nowosielski:** writing – review and editing. **Marcin A. Jedryka:** writing – review and editing. **David Hardisson:** writing – review and editing. **Stefano Restaino:** writing – review and editing. **Sonia Gatius:** writing – review and editing. **Zoltan Novak:** writing – review and editing. **Amaia Sagasta Lacalle:** writing – review and editing. **Susana Lopez:** writing – review and editing. **Martin Pešta:** investigation, methodology, writing – review and editing. **Marcin Zebalski:** investigation. **Piotr Lepka:** investigation. **María Dolores Diestro:** investigation, writing – review and editing. **Giuseppe Vizzielli:** investigation, writing – review and editing. **Xavier Matias‐Guiu:** writing – review and editing. **Tímea Echim:** investigation. **Emma Natalia Camacho Urkaray:** investigation. **Iván Rienda:** investigation. **Robert Slunečko:** investigation, methodology. **Andrzej Czekanski:** investigation. **Alberto Berjón:** investigation. **Laura Mariuzzi:** investigation. **Ana Velasco:** investigation. **Judit Betenbuk:** investigation. **Isabel Guerra Merino:** investigation. **Pablo Padilla‐Iserte:** investigation. **Petr Stráník:** investigation. **Vendula Smoligová:** investigation. **Jiří Presl:** supervision, conceptualization.

## Ethics Statement

The study was approved by the University Hospital and Medical Faculty in Pilsen ethical committee (ID 66/2020). Ethical committee approvals were obtained by the relevant review board for each of the collaborating centers.

## Consent

All patients gave their written informed consent for inclusion before they participated in the study.

## Conflicts of Interest

The authors declare no conflicts of interest. Although this study received funding support from Sysmex Europe GmbH, the manufacturer of the OSNA system, the sponsor had no role in the design of the study, data collection, analysis or interpretation. All aspects of the study were independently conducted by the academic investigators to ensure scientific integrity and objectivity.

## Data Availability

The data that support the findings of this study are available from the corresponding author upon reasonable request.
